# Clinical impact of LH rises prior to and during ganirelix treatment started on day 5 or on day 6 of ovarian stimulation

**DOI:** 10.1186/1477-7827-11-90

**Published:** 2013-09-12

**Authors:** John L Frattarelli, Torbjörn Hillensjö, Frank J Broekmans, Han Witjes, Jolanda Elbers, Keith Gordon, Bernadette Mannaerts

**Affiliations:** 1 Advanced Reproductive Medicine & Gynecology of Hawaii, Inc., 1401 South Beretania St, Honolulu, Hawaii 96814, USA; 2 Fertility Center Scandinavia, Carlanderska Hospital, Göteborg, Sweden; 3 Department of Reproductive Medicine, University Medical Center Utrecht, Utrecht, Netherlands; 4 MSD Oss B.V., Oss, Netherlands; 5 Merck Sharp & Dohme Corp., Whitehouse Station, NJ, USA

**Keywords:** Ovarian stimulation, GnRH antagonist, Serum LH, Ovarian response, Ongoing pregnancy rate

## Abstract

**Background:**

We sought to evaluate the incidence and clinical impact of luteinizing hormone (LH) rises prior to and during gonadotropin-releasing hormone (GnRH) antagonist treatment started on day 5 or 6 of ovarian stimulation with recombinant follicle-stimulating hormone (rFSH).

**Methods:**

Pooled data from three trials with the GnRH antagonist ganirelix started on day 5 (n = 961) and from five trials with ganirelix started on day 6 (n = 1135) of ovarian stimulation with rFSH were retrospectively analyzed.

**Results:**

The incidence of LH rises (LH ≥ 10.0 IU/L) prior to ganirelix treatment was 2.3% and 6.6% on ganirelix start days 5 and 6, respectively (P < 0.01). During ganirelix treatment this incidence was 1.2% and 2.3%, respectively (P = 0.06). Women with LH rise on day 5 or 6 had a higher ovarian response with more oocytes recovered, mean ± SD, 12.9 ± 8.5 versus no LH rise, 10.2 ± 6.4 (P < 0.01). In women with and without LH rise prior to ganirelix treatment the ongoing pregnancy rates were similar (26.0% vs 29.9%; odds ratio [OR], 0.89; 95% confidence interval [CI], 0.55-1.44). Women with LH rise during ganirelix treatment had a lower ovarian response with 7.5 ± 6.7 oocytes recovered versus no LH rise, 10.2 ± 6.4 (P = 0.02) and a tendancy for a lower chance of ongoing pregnancy (16.7% vs 29.9%; OR, 0.52; 95% CI, 0.21-1.26).

**Conclusions:**

The incidence of early and late LH rises was low but may be further reduced by initiating ganirelix on stimulation day 5 rather than on day 6. In contrast to women with an early LH rise, women with a late LH rise may have a reduced chance of ongoing pregnancy.

## Background

In the early follicular phase of the natural cycle, high follicle-stimulating hormone (FSH) concentrations initiate follicular development, which leads to rising serum estradiol concentrations. This, in turn, causes a negative feedback in FSH release from the pituitary, resulting in atresia of the smaller follicles and selection of a single dominant follicle. Once serum estradiol concentrations surpass a certain level, a positive feedback loop stimulates the pituitary and results in the preovulatory luteinizing hormone (LH) surge. This LH surge is responsible for final oocyte maturation and subsequent ovulation [1].

During induced multiple follicular development, when FSH concentrations are maintained because of exogenous gonadotropin administration, natural selection of a single dominant follicle does not occur and multiple follicles continue to grow. This increased number of follicles produces higher serum estradiol concentrations and consequently, the serum estradiol concentration that triggers the preovulatory LH surge is reached prematurely, i.e., before the follicles have fully developed. For successful assisted reproduction treatment, it is essential to prevent premature luteinization and ovulation. Without intervention, premature luteinization occurs in about 25% of ovarian stimulation cycles, leading to cycle cancellation or compromised treatment outcomes [2,3]. Premature luteinization may have an unfavorable impact on oocyte quality, fertilization, and implantation. Use of a gonadotropin-releasing hormone (GnRH) agonist [4] or antagonist [5] has been shown to improve the reproductive outcome of ovarian stimulation by preventing premature LH surges.

GnRH antagonists first became available for the prevention of premature endogenous LH surges in women undergoing ovarian stimulation a decade ago. However, late LH rises occasionally occur in women during GnRH antagonist treatment, sometimes due to drug noncompliance [6] or possibly due to increased endogenous GnRH release in response to rising serum estradiol concentrations. If these LH rises are considerable and occur with premature progesterone (P) rises, ovulation becomes imminent.

Women with induced multifollicular development may also have an early LH rise prior to the start of the GnRH antagonist; this is more frequently observed in high responders. In earlier clinical studies, the GnRH antagonist ganirelix was often fixed to start on stimulation day 6; however, in high ovarian responders it may be preferred to start ganirelix treatment on day 5 to lower the incidence of early LH rises. The latter is determined by the study population, the gonadotropin of choice, and the FSH starting dose. The incidence of early LH rises on day 6 of stimulation prior to the first ganirelix administration was 15% when the starting dose of recombinant FSH (rFSH) was 225 IU [7] and 4.3% when the starting dose of rFSH was 150 IU [8]. The present study was undertaken to evaluate the incidence of LH rises and their clinical impact when occurring prior to ganirelix treatment started on either day 5 or 6 or during ganirelix treatment in more than 2000 patients following ovarian stimulation with recombinant FSH.

## Methods

### Clinical trials with ganirelix started on day 5 of ovarian stimulation

Individual patient data were pooled from three multicenter, randomized clinical trials with a fixed ganirelix (Ganirelix Acetate Injection, Orgalutran, N.V. Organon, The Netherlands) treatment (0.25 mg/d) start on the morning of day 5. All three trials were primarily designed to show efficacy and safety of corifollitropin alfa in ovarian stimulation versus rFSH. Only information obtained from the rFSH reference arms of these three trials was included in this analysis.

i. The corifollitropin alfa dose-finding study - a phase 2 open-label trial comprising three corifollitropin alfa treatment groups and an rFSH reference group [9]. Women aged 20–39 years with a body mass index (BMI) of 17–31 kg/m^2^ received a fixed daily dose of 150 IU rFSH from cycle days 2 or 3 up to the day of human chorionic gonadotropin (hCG) administration (N = 82).

ii. Engage - a phase 3 double-blind efficacy and safety study that compared 150 μg corifollitropin alfa with daily 200 IU rFSH [10]. Women aged 18–36 years with a BMI of 18–32 kg/m^2^ received a fixed daily dose of 200 IU rFSH up to and including the day of hCG administration (N = 750).

iii. Ensure - a phase 3 double-blind efficacy and safety study that compared 100 μg corifollitropin alfa with daily 150 IU rFSH [11]. Women aged 18–36 years with a BMI of 18–32 kg/m^2^ received a fixed daily dose of 150 IU rFSH up to and including the day of hCG administration (N = 129).

### Clinical trials with ganirelix started on day 6 of ovarian stimulation

Individual patient data from five clinical trials with a fixed ganirelix start on the morning of day 6 of ovarian stimulation with rFSH were pooled. Only data using the approved dose of ganirelix (0.25 mg/d up to the day of hCG administration) were used for the analyses.

i-iii. Three pivotal phase 3 randomized controlled trials to assess the efficacy, safety, and tolerability of ganirelix. Women aged 18–39 years with a BMI of 18–29 kg/m^2^ received daily rFSH from stimulation day 1 up to the day of hCG administration, with dose adjustment as necessary after stimulation day 6.

Start dose of rFSH 150 IU/d, N = 462 [8]

Start dose of rFSH 225 IU/d, N = 197 [7]

Start dose of rFSH 150 IU/d, N = 226 [12].

iv. A single-center, open-label phase 3 safety trial undertaken in Israel between March 1998 and July 1999 [13], start dose of rFSH 150 IU/d, N = 167.

v. A multicenter, open-label, randomized, pharmacokinetic and pharmacodynamic study carried out between December 2000 and November 2001 [14], start dose of rFSH 150 IU/day, N = 83.

### Assessments

The size and number of follicles was measured by ultrasound scan.

Validated immunoassays were performed to measure serum concentrations of LH, estradiol, and P. In all trials (except the North American Ganirelix Study) these measurements were carried out by one central laboratory (Waltrop, Germany) using a time-resolved fluoroimmunoassay (AutoDelfia immunofluorometric assay, PerkinElmer Life and Analytical Sciences, Brussels, Belgium). Detection limits for serum LH, estradiol, and P were 0.6 IU/L, 50 pmol/L, and 1.3 nmol/L (0.4 ng/mL), respectively. In the North American Ganirelix Study, serum LH concentrations were measured by the Immulite 1000 LH assay (DPC, Los Angeles, CA) and estradiol and P concentrations at a central laboratory (Quest Diagnostics, USA).

LH rise prior to and during ganirelix treatment was defined as serum LH ≥10 IU/L and P rise was defined as serum P ≥3.18 nmol/L (1 ng/mL).

### Statistical analysis

Individual patient data from trials in which ganirelix was started on stimulation day 5 were pooled (N = 961) and, separately, the data from trials in which ganirelix was started on day 6 were pooled (N = 1135) as if the data were from one large trial. The data used in the current analyses reflect minor corrections to the number of oocytes retrieved previously published in the Engage [10] and Ensure [11] trial data.

Descriptive statistics of demographic, baseline, and treatment characteristics were presented by start day of ganirelix treatment (day 5, day 6).

The incidence of LH rises with or without P rises prior to ganirelix treatment (early rises) and during ganirelix treatment (late rises) were presented by start day of ganirelix treatment. The incidences between day 5 and day 6 ganirelix start were compared and tested using a Fisher's exact test.

As the overall incidence of LH rises is low, individual patient data from all trials were pooled (N = 2096) to explore the effect of early and late LH rises on estradiol concentrations, number of follicles and oocytes, and ongoing pregnancy rates.

Direct comparisons for the ovarian response and ongoing pregnancy rates between women with an LH rise prior to and during ganirelix treatment and women who had no LH rise were performed and included all trials for both groups. However, such comparison was not performed for subjects who started ganirelix on day 5 and on day 6 because these data sets were from different clinical trials conducted during different time periods and in different geographic regions, which may affect these clinical outcomes [15].

Analysis of variance (ANOVA) was applied to estimate the effect of early and late LH rises on the number of follicles ≥11 mm on day of hCG and on the number of oocytes retrieved, respectively. Factor trial was included as an independent factor (covariate) in the ANOVA model to adjust for trial effects. The effect of LH rise on the median estradiol concentrations was estimated using the Wilcoxon rank sum test. Logistic regression analysis was applied to estimate the odds ratio (OR) for ongoing pregnancy between women with early LH rise and no LH rise and between women with late LH rise and no LH rise, respectively. Trial was included as a covariate in the logistic model.

## Results

### Patient demographic and baseline characteristics

This analysis included 2096 women who started stimulation on cycle day 2 or 3; 961 of these women started ganirelix treatment on stimulation day 5 and 1135 started ganirelix treatment on stimulation day 6. The demographic and baseline characteristics for these women are given in Table 1. Women who started ganirelix treatment at stimulation day 5 had a similar age, body weight, and BMI as patients who started ganirelix treatment at stimulation day 6. The mean (±SD) age of women with an LH rise prior to ganirelix treatment or with an LH rise during ganirelix treatment, or without any LH rise was 31.8 ± 3.7, 31.4 ± 3.8, and 31.5 ± 3.8, respectively. Women who started ganirelix treatment at stimulation day 5 or 6 had the same infertility history although their geographic regions were clearly different.

**Table 1:** Demographic and baseline characteristics

	**Ganirelix started on day 5 (n = 961)**	**Ganirelix started on day 6 (n = 1135)**
*Age, y, mean ± SD*	31.5 ± 3.3	31.4 ± 4.1
*Body weight, kg, mean ± SD*	66.2 ± 8.6	63.5 ± 10.2
*BMI, kg/m*^*2*^*, mean ± SD*	24.1 ± 3.0	23.4 ± 3.3
*Region, %*		
*North America*	41.9	17.4
*Asia*	5.9	0
*Middle East*	0	24.4
*Europe*	52.1	58.2
*Duration of infertility, y, mean ± SD*	3.2 ± 2.2	4.6 ± 3.2
*Primary infertility, %*	54.0	57.8
Cause of infertility^*^, %		
*Male factor*	47.7	51.9
*Tubal factor*	24.3	32.2
*Endometriosis*	14.0	8.7
*Cervical mucus problems*	0.6	1.2
*Unexplained infertility*	28.8	13.7
*Other*	6.0	4.1

*BMI* body mass index, *SD* standard deviation.

^*^A patient could have more than one cause of infertility.

### Treatment details

The median duration of ganirelix treatment was 5.0 days both for women who started ganirelix treatment on stimulation day 5 and for women who started treatment on stimulation day 6 (percentiles P5, P95: 3.0, 7.0 for start day 5, and 2.0, 9.0 for start day 6). Women who started ganirelix on day 5 received most frequently a daily starting dose of 200 IU rFSH (78%) whereas women who started ganirelix on day 6, received most frequently a daily starting dose of 150 IU rFSH (69%).

When a higher rFSH starting dose was applied, the duration of stimulation was shorter, leading to a similar overall amount of rFSH used in both groups. The median (P5, P95) total dose of rFSH was 1600 IU (1200, 2200) and 1575 IU (1050, 3000) in the group starting day 5 and day 6, respectively.

### Incidence of LH rise

The incidence of LH rises measured at stimulation day 5 or 6 prior to the start of ganirelix treatment (early LH rises) and during ganirelix treatment (late LH rises) is presented in Table 2.

**Table 2:** Incidence of LH rises (≥10 IU/L) and of LH rises with P rises (≥3.18 nmol/L) measured at stimulation day 5 or 6 prior to the start of ganirelix treatment and during ganirelix treatment

		**Ganirelix start day**	**% (95% CI), n/N**	P value*
*LH rises*	Early	Day 5	2.3 (1.4-3.4)	< 0.01
			22/955	
		Day 6	6.6 (5.2-8.2)	
			74/1113	
	Late	Day 5	1.2 (0.6-2.0)	0.06
			11/949	
		Day 6	2.3 (1.4-3.3)	
			25/1096	
*LH+P rises*	Early	Day 5	1.0 (0.5-1.9)	0.11
			10/955	
		Day 6	2.0 (1.2-2.9)	
			22/1113	
	Late	Day 5	0.5 (0.2-1.2)	0.44
			5/949	
		Day 6	0.9 (0.4-1.6)	
			10/1096	

Early indicates on start day of ganirelix treatment; Late indicates during ganirelix treatment. *CI* confidence intervals, *LH* luteinizing hormone, *P* progesterone. *Day 5 versus day 6.

The incidence of early LH rises was 2.3% (95% confidence interval [CI], 1.4-3.4) in women who started ganirelix treatment on stimulation day 5 and 6.6% (95% CI, 5.2-8.2) in women who started ganirelix on stimulation day 6 (P < 0.01). The incidence of early LH rises with concomitant P rises on days 5 and 6 was 1.0% (95% CI, 0.5-1.9) and 2.0% (95% CI, 1.2-2.9), respectively.

The incidence of late LH rises in women who started ganirelix treatment at stimulation day 5 was 1.2% (95% CI, 0.6-2.0) and in women who started ganirelix at stimulation day 6 was 2.3% (95% CI, 1.4-3.3) (P = 0.06). The incidence of late LH rises with concomitant P rises on days 5 and 6 was 0.5% (95% CI, 0.2-1.2) and 0.9% (95% CI, 0.4-1.6), respectively.

### Ovarian response according to LH rise

Table 3 shows the ovarian response in women with early LH rise, late LH rise, and no LH rise. Five subjects had an early and a late LH rise. Women with an early LH rise prior to ganirelix treatment had a higher mean number of follicles ≥11 mm on the day of hCG than women without an LH rise (P < 0.01). Accordingly, their median serum estradiol concentration on the day of hCG was also higher.

**Table 3:** Ovarian response and clinical outcome according to early or late LH rise versus no LH rises

	**Early LH rises (n = 96)**	**Late LH rises (n = 36)**	**No LH rises (n = 1962)**
*Follicles ≥11 mm on day of hCG, mean ± SD*	13.2 ± 7.8	8.9 ± 6.6	11.6 ± 6.1
*Serum estradiol on day of hCG, pmol/L, median (P5, P95)*	7303 (3171, 16478)	5391 (573, 15047)	4367 (1369, 10955)
*Oocytes retrieved, mean ± SD*	12.9 ± 8.5	7.5 ± 6.7	10.2 ± 6.4
*Ongoing pregnancy rate, %*	26.0	16.7	29.9

*hCG* human chorionic gonadotropin, *P5*, *P95* 5th and 95th percentiles, *SD* standard deviation.

Women with a late LH rise during ganirelix treatment had a lower mean number of growing follicles on the day of hCG administration than women without an LH rise (P = 0.01). Their median estradiol concentration was not significantly different from those with no LH rise (P = 0.38).

### Clinical outcome according to LH rise

Table 3 shows the clinical outcome in women with early LH rise, late LH rise, and no LH rise. In women with an early LH rise prior to ganirelix treatment more oocytes were retrieved compared with women without an LH rise (P < 0.01). The ongoing pregnancy rate following fresh embryo transfer was similar in women with an early LH rise and no LH rise. The estimated odds ratio (OR [95% CI]) for ongoing pregnancy, adjusted for trial effect, for women with an early LH rise versus women without an LH rise was 0.89 (0.55-1.44).

In women with a late LH rise during ganirelix treatment, fewer oocytes were retrieved compared with women without an LH rise (P = 0.02). The ongoing pregnancy rate following fresh embryo transfer was numerically lower in women with a late LH rise than in those with no LH rise. The estimated OR (95% CI) for ongoing pregnancy, adjusted for trial effect, for women with a late LH rise versus women without an LH rise was 0.52 (0.21-1.26). In total, 15 women had an LH and P rise during ganirelix treatment and five of these 15 women still became pregnant.

## Discussion

This retrospective pooled analysis indicates that the incidence of early and late LH rises prior to and during ganirelix treatment is low and comparison of the clinical outcome shows that, in contrast to women with an early LH rise, women with a late LH rise may have a reduced chance of ongoing pregnancy.

The incidence of LH rises on stimulation day 5 was significantly lower than on day 6 and the incidence during ganirelix treatment tended to be lower in subjects who started treatment on day 5 rather than on day 6, indicating that an earlier initiation of ganirelix treatment may further lower the incidence during ganirelix treatment as observed in this study. The limitation of the retrospective approach of this study is acknowledged.

Women with or without LH rises had the same average age; thus, it may be difficult to predict which women will have an increased risk for a premature LH rise. Prediction of ovarian response may be facilitated by pretreatment assessment of the antral follicle count and of anti-Müllerian hormone, both of which have been shown to be reliable predictors of ovarian response [16,17]. When women start ovarian stimulation with a relatively high dose of rFSH they may subsequently present with more follicles and higher serum estradiol concentrations during the early follicular phase, thereby reaching the threshold for LH surge earlier during stimulation. In a previous study in which women were treated with a fixed starting dose of 225 IU FSH, employment of an algorithm of criteria to prevent premature LH surges resulted in GnRH antagonist initiation earlier than stimulation day 6 in two thirds of patients (138/208 initiated on days 4 or 5). This earlier start resulted in an improved clinical outcome [18].

In contrast to previous publications that have reported premature LH peaks in up to 22% of subjects during (mild) stimulation while treated with a GnRH antagonist [19-21], the current analysis indicates that LH rise prior to and during ganirelix treatment is a comparatively infrequent event in women aged up to 39 years with regular menstrual cycles undergoing ovarian stimulation prior to in vitro fertilization or intracytoplasmic sperm injection. In addition, the incidence of a concomitant rise in P was even lower, which for the early LH rises may be related to the low number of LH receptors during the first days of stimulation [22].

While women with an early LH rise prior to ganirelix treatment had higher numbers of oocytes retrieved compared with women without an LH rise, the impact of this early LH rise seems limited as the chance of ongoing pregnancy was not compromised. This may be explained by a possible short-lived pulse of LH rather than a precursor to luteinization and premature ovulation. In contrast, women with a late LH rise during ganirelix treatment had a lower ovarian response and had a lower chance of ongoing pregnancy, though the OR for ongoing pregnancy for women with an LH rise versus women without LH rise was not significant. Some of these LH rises may have been caused by drug noncompliance; however, these cannot be documented because ganirelix concentrations were not measured during these trials. Other LH rises may indeed be related to either premature luteinization or a low ovarian response, and the causes require further investigation.

While a daily dose of 0.25 mg ganirelix has been demonstrated to be an effective dose to prevent premature LH rises during multiple follicular development in in vitro fertilization cycles, there is some evidence that a GnRH antagonist is less effective in blocking the positive feedback effect of estradiol in women with mild or nonstimulated ovaries [23,24]; this is in agreement with the lower ovarian response of women with an LH rise during ganirelix treatment in the current retrospective analysis. The cause of these LH rises remains to be elucidated but it has been suggested that a reduced production of gonadotropin surge-attenuating factor may be involved, which is hypersecreted in the case of induced multiple follicular development [23].

Late LH rises during GnRH antagonist treatment lower the chance of pregnancy and may be further reduced by starting antagonist treatment timely. These results support a flexible regimen of starting ganirelix treatment on day 5 or day 6 of stimulation, depending on the ovarian response.

## Conclusions

This combined retrospective analysis indicates that the incidence of LH rises prior to and during ganirelix treatment is low and may be further reduced by initiating ganirelix on ovarian stimulation day 5 rather than on day 6. An early LH rise prior to ganirelix treatment may occur more frequently in women with a relatively high ovarian response, whereas a late LH rise during ganirelix treatment occurs most commonly in women with a normal to low ovarian response. In contrast to women with an early LH rise, women with a late LH rise may have a reduced chance of ongoing pregnancy.

## Competing interests

JLF has received support for travel to meetings from Merck & Co., Inc., Whitehouse Station, NJ. FJB has received a grant to his institution from College voor Ziektekosten Verzekeringen (Organisation of Health Insurance Policies). HW, JE, and BM are employees of MSD Oss B.V., Oss, The Netherlands. KG is an employee of Merck & Co., Inc., Whitehouse Station, NJ. TH has no competing interests.

## Authors’ contributions

JLF, TH, FJB, HW, JE, KG and BM took part in the analysis and interpretation of data, writing the manuscript and in the final approval of the version to be published. All authors read and approved the final manuscript.
